# Plasma HMGB-1 Levels in Subjects with Obesity and Type 2 Diabetes: A Cross-Sectional Study in China

**DOI:** 10.1371/journal.pone.0136564

**Published:** 2015-08-28

**Authors:** Hang Wang, Hua Qu, Huacong Deng

**Affiliations:** M.D. Department of Endocrinology, The First Affiliated Hospital of Chongqing Medical University, Chongqing, China; University of Catanzaro Magna Graecia, ITALY

## Abstract

**Object:**

To detect the levels of plasma High-Mobility Group Box-1(HMGB1) in Chinese subject with obesity and type 2 diabetes mellitus (T2DM), and to investigate the correlations between plasma HMGB1 concentration and parameters of body fat, insulin resistance (IR) metabolism and inflammation.

**Methods:**

This study recruited 79 normal glucose tolerance (NGT) subjects and 76 newly diagnosed T2DM patients. NGT and T2DM groups were divided into normal weight (NW) and obese (OB)subgroups respectively. Anthropometric parameters such as height, weight, waist circumference, hip circumference and blood pressure were measured. Plasma concentrations of HMGB1, IL-6, fasting plasma glucose (FPG), 2 hours post challenge plasma glucose (2hPG), serum lipid, glycated hemoglobin (HbA_1C_) and fasting insulin (FINS) were examined. The homeostasis model assessment (HOMA) was performed to assess IR status.

**Results:**

Plasma HMGB1 levels were higher in T2DM group than that in NGT group. The concentrations of serum HMGB1 were also higher in subjects with OB than those in subjects with NW both in NGT and T2DM groups. Plasma levels of HMGB1 were positively correlated with waist hip ratio (WHR), blood pressure, FPG, FINS, HOMA-IR, TG, IL-6 and negatively correlated with HOMA-βand high-density lipoprotein-cholesterol (HDL-c) independent of age, gender and BMI. Plasma levels of HMGB1 were significantly correlated with diabetes in fully adjusted models.

**Conclusion:**

Plasma HMGB1 levels were increased in Chinese subjects with pure T2DM, which might be caused by IR. Serum HMGB1 participated in the pathological process of obesity and T2DM via its proinflammatory effect.

## Introduction

Many nuclear proteins, in addition to their nuclear actions,can also be released into the extracellular milieuand participate in the pathology of various diseases. These proteins are activated in response to multiple stimulations and then trigger or contribute to the development of disease. High-Mobility Group Box-1(HMGB1) is part of the family of the High-Mobility Group Box proteins. It is a highly conserved protein that is present in the nucleus of almost all cell types to maintain the nucleosome structure and regulate gene transcription. Recent researchhas demonstrated that HMGB1 can be secreted into the extracellular milieu to function as a proinflammatory cytokine[1]. Meanwhile, increasing numbers of studies indicate that type 2 diabetes mellitus (T2DM) and obesity are not merely metabolic diseases but are also related to inflammatory dysfunction[2–3]. AlthoughHMGB1 is reportedly associated with obesity in adolescents[4], research has rarely provided direct evidence to further confirm the relationship between T2DM,obesity and HMGB1. The current study is aimed to investigate the relationship of plasma HMGB1 levels with body fat, glycolipid metabolism, insulin resistance (IR) and inflammation in obese adults and newly diagnosed T2DM patients.

## Materials and Methods

### Subjects

The trial was implemented from February 2014 to December 2014. A total of 328 volunteers were recruited from outpatients of the first affiliated hospital of Chongqing medical university. Of 328 participants, 94 were excluded due to correlated datamissed. The glucose status of all participants were verified by75g oral glucose tolerance test (OGTT), then according to the diagnostic criteria of World Health Organization (WHO) in 1999[5], all volunteers were diagnosed T2DM or normal glucose tolerance (NGT). Of 234 volunteers, 79 impaired glucose tolerance (IGT)subjects were excluded. The NGT group contained 79 subjects and the T2DM group included 76 newly diagnosed T2DM patients. Newly diagnosed T2DM was defined as none of the patients received any glycemic control treatment such as diet, exercise and medication before. Then every volunteer received physical examination. A structured questionnaire that collected information on demographics, medications, self-reported medical history, and lifestyle was administered face to face by trained interviewers. According to the obesity criteria of WHO-Western Pacific Region (2000) that BMI ≥ 25 kg/m^2^ was diagnosed as obesity[6], all participants were divided into four subgroups: NGT-normal weight (NGT-NW) subgroup, NGT-obesity (NGT-OB) subgroup, T2DM-NW subgroup and T2DM-OB subgroup. Exclusion criteria of volunteers included current or past smoking history and excessive alcohol consumption, defined as average daily consumption of alcohol >20 g/day (140 g/week) in males and > 10 g/day (70 g/week) in females based on self-reported frequency and daily amount of alcohol consumption, acute and chronic complications of diabetes, stage 2 hypertension (resting blood pressure≥160/100 mmHg), history of cardiovascular disease (myocardial infarction, unstable angina, stroke, peripheral artery disease, or cardiovascular revascularization), acute and chronic inflammatory diseases (determined by clinical symptom of infection, or blood leukocyte >7 ×10^9^/L, or those taking medications that could affect their inflammatory status within 3 months), hepatic or renal disease, and systemic corticosteroid treatment. Women who were pregnant, breastfeeding or taking acyeterion were also excluded from this study. The study was approved by the Ethical Committee of Chongqing Medical University. Signed informed consents were obtained from all participants in this study.

### Clinical and biochemical evaluations

#### Physical examination

Height, weight, waist circumference, and hip circumference were measured by certified technicians according to a standardized protocol. Body mass index (BMI) was computed as weight divided by squared height (kg/m^2^), waist to hip ratio (WHR) was calculated as waist circumference divided by hip circumference. After an initial 5-min rest, BP was measured 3 times at 1-min interval using an Omron Model HEM-725 FUZZY random-zero sphygmomanometer (Omron Company, Dalian, China). All BP measurements were averaged.

#### Biochemical examination

Each subject received OGTT. Blood samples were collected in the morning after an overnight fast of ≥ 8 hours. Plasma samples were obtained by centrifugation at 4°C and kept at -80°C until used in this study within 3 month period. Fasting plasma glucose (FPG) and 2-hour plasma glucose (2hPG) were assayed by a glucose oxidase method. Glycated hemoglobin (HbA1C) was measured by isoelectric focusing. The levels of triglyceride (TG), total cholesterol (TC), high-density lipoprotein cholesterol (HDL-c), and low-density lipoprotein cholesterol (LDL-c) were detected by biochemical auto analyzer (Beckman CX-7 Biochemical Autoanalyser, Brea, CA, USA). Fasting insulin (FINS) was measured in serum by RIA using human insulin as standard (Linco, St Charles, MO, USA). The homeostasis model assessment (HOMA) of IR was computed as: HOMA-IR = FINS (mU/L) × FPG (mmol/L) /22.5, homeostasis model assessment (HOMA) of β was calculated as: HOMA-β = 20 ×FINS (mU/L) / (FPG (mmol/L)– 3.5).

### Assessment of plasma HMGB1 and interleukin- 6 (IL-6) levels

Plasma levels of HMGB1 and IL-6 were determined by enzyme-linked immunosorbent assays according to the manufacturers’ instructions(Human ELISA kit, Uscn Life Science Inc, Wuhan, China). All samples were run in duplicate and repeated if there was a >15% difference between duplicates. Intra-assay coefficient of variance (CV) was 10% and inter-assay CV of 12%. No significant cross-reactivity or interference was observed.

### Statistical method

Data were expressed as mean ± standard deviation (SD). Continuous variables in skewed distribution were log-transformed for statistical analysis. An independent-samples t tests were used to compare continuous variables between the 2 groups. Analysis of variance (ANOVA) and Student-Newman-Keuls tests were performed for multiple and pairwise comparisons, respectively. Interrelationships between variables were analyzed by Spearman’s partial correlation test and multivariate logistic regression analyze. All calculations were performed by SPSS 19.0 (SPSS Inc., Chicago, US) for Windows. *P* was obtained from 2-sided tests (significance < 0.05).

## Results

### The clinical characteristics were described in Table 1

**Table 1:** Clinical and laboratory characteristics of the study subjects.

	NGT		T2DM			
variables	NGT-NW (n = 39)	NGT-OB (n = 36)	T2DM-NW (n = 40)	T2DM-OB (n = 40)	χ2 / F	P
Sex (M/F)	39 (22 / 17)	36 (16 / 20)	40 (17 / 23)	40 (21 / 19)	2.028	0.567
Age(year)	58.54±8.25	61.55±8.87^d^	65.56±7.49^c^ ^,^ ^d^	64.08±6.37^d^	1.193	0.242
BMI(kg/m^2^)	22.11±2.05	26.93±1.62^d^ ^,^ ^e^	22.21±1.71	28.90±2.72^d^ ^,^ ^e^ ^,^ ^f^	103.794	<0.001
WC(cm)	79.38±7.10	87.46±6.70^d^ ^,^ ^e^	80.73±7.70	93.34±13.22^d^ ^,^ ^e^ ^,^ ^f^	19.272	<0.001
WHR	0.86±0.05	0.88±0.05	0.88±0.07	0.94±0.06^d^ ^,^ ^e^ ^,^ ^f^	12.700	<0.001
SBP (mm Hg)	119.08±10.89	119.75±10.77^e^	130.32±13.58^d^	141.74±17.38^d^ ^,^ ^e^ ^,^ ^f^	24.085	<0.001
DBP (mm Hg)	71.15±6.52	73.25±7.03	74.59±9.05	81.05±11.79^d^ ^,^ ^e^ ^,^ ^f^	8.979	<0.001
FPG (mmol/L)	5.22±0.93	5.29±0.25^e^	6.69±1.98^d^	6.96±1.33^d^ ^,^ ^f^	19.171	<0.001
2hPG (mmol/L)	6.36±0.91	6.07±1.06^e^	12.94±3.21^d^	12.54±3.27^d^ ^,^ ^f^	92.710	<0.001
HbA_1C_ (%)	5.59±0.35	5.55±0.52^e^	6.31±1.25^d^	6.39±0.75^d^ ^,^ ^f^	12.335	<0.001
FINS (mU/L)	5.40±2.11	7.06±2.17^a^	7.53±4.37^a^	11.21±4.79^d^ ^,^ ^e^ ^,^ ^f^	18.145	<0.001
HOMA-IR	1.27±0.57	1.66±0.52	2.14±1.62^d^	3.45±1.56^d^ ^,^ ^e^ ^,^ ^f^	24.503	<0.001
HOMA-β	97.57±19.94	81.68±22.10^d^ ^,^ ^b^	70.84±21.15^d^	57.71±20.44^d^ ^,^ ^e^ ^,^ ^f^	25.708	<0.001
HDL-c (mmol/L)	1.42±0.20	1.32±0.26	1.35±0.35	1.20±0.27^d^ ^,^ ^b^	4.318	0.006
LDL-c (mmol/L)	2.87±0.74	2.97±0.88	2.68±1.11	2.78±0.80	0.697	0.555
TC (mmol/L)	4.98±0.93	5.02±1.05	4.79±1.30	4.88±0.95	0.364	0.779
TG (mmol/L)	1.34±0.67	1.60±0.74	1.74±0.91	2.33±1.83^d^ ^,^ ^b^ ^,^ ^f^	5.136	0.002
IL-6(pg/mL)	58.92±16.25	80.39±24.13^d^	86.07±18.61^d^	103.88±34.58^d^ ^,^ ^e^ ^,^ ^f^	5.787	0.001

Data are presented as means±SD. NGT, normal glucose tolerance; T2DM, type 2 diabetes mellitus; NW, normal weight; OB, obesity; BMI, body mass index; Wc, waist circumference; WHR, waist hip ratio; SBP, systolic blood pressure; DBP, diastolic blood pressure; FPG, fasting plasma glucose; 2hPG, 2 hours postchallenge plasma glucose; HbA_1C_, glycated hemoglobin; FINS, fasting serum insulin; HOMA-IR, Homeostasis Model Assessment for insulin resistance; HOMA-β, Homeostasis Model Assessment for beta-cell function; TC, total cholesterol; TG, triglyceride; HDL-c, high-density lipoprotein-cholesterol; LDL-c, low-density lipoprotein-cholesterol; IL-6, interleukin- 6.

^a^P<0.05 compared with NGT-NW,

^b^P<0.05 compared with T2DM-NW,

^c^P<0.05 compared with NGT-OB,

^d^P<0.01 compared with NGT-NW,

^e^P<0.01 compared with T2DM-NW,

^f^P<0.01 compared with NGT-OB.

There were no significant differences among the four subgroups in variables including gender, age, LDL-c and TC. In comparison to the other three subgroups, participants with diabetes and obesity (T2DM-OB) had a higher levels of BMI,WHR, SBP,DBP, FINS, HOMA-IR, TG and IL-6 (all *P*<0.01), whereas HOMA-β was significantly decreased (*P*<0.05 or 0.01), indicating that both diabetes and obesity affected the metabolization-related biomarkers and inflammation. Besides, higher glycemia indicators including FPG, 2hPG and HbA_1C_ were observed in T2DM group in contrast to the NGT group (all *P*<0.01), and obese participants with/without T2DM had a obvious higher level of BMI.

### Plasma HMGB1 levels were presented in Fig 1

**Fig 1:**
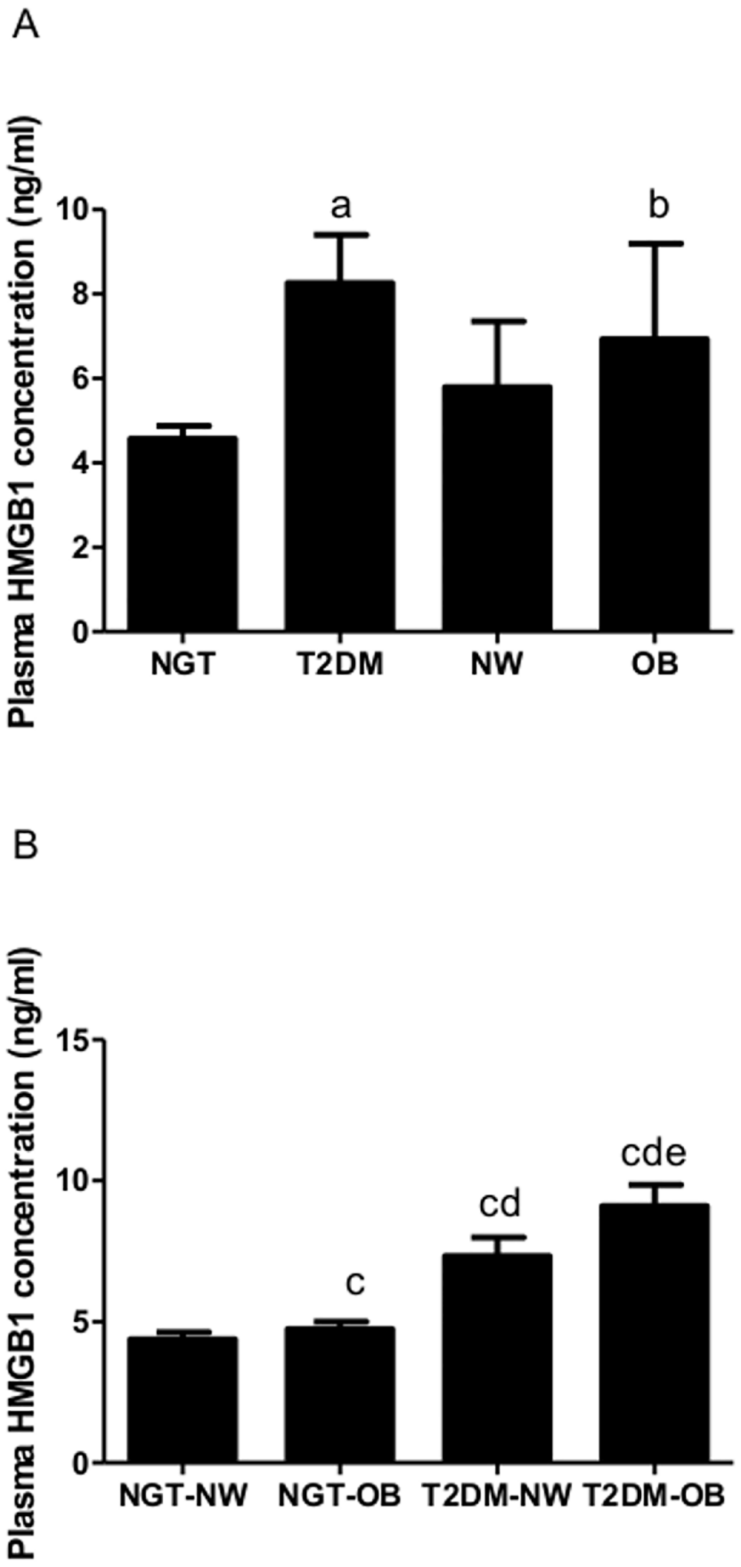
Plasma HMGB1 concentrations. A. Comparison of HMGB1 concentrations in NGT, T2DM, NW and OB groups. B. Comparison of HMGB1 concentrations in NGT-NW, NGT-OB, T2DM-NW and T2DM-OB subgroups. aP < 0.001 compared with NGT. bP < 0.001 compared with NW. cP < 0.01 compared with NGT-NW. dP < 0.001 compared with NGT-OB. eP < 0.001 compared with T2DM-NW.

Between NGT and T2DM groups, the plasma levels of HMGB1 were significantly increased in T2DM than those in NGT (8.27 ± 1.14 vs 4.57 ± 0.31, *P*< 0.001), while between NW and OB groups, the plasma levelsof HMGB1 were significantly increased in OB than those in NW (6.93 ± 2.26 vs 5.80 ± 1.56, *P*< 0.001). Among NGT-NW, NGT-OB, T2DM-NW and T2DM-OB subgroups, the plasma HMGB1 levels in NGT-OB were higher than those in NGT-NW (4.95 ± 0.76 vs 4.44 ± 0.25, *P*< 0.01), while the plasma HMGB1 concentrations in T2DM-OB were higher than those in T2DM-NW (9.11 ± 0.75 vs 7.43 ± 0.81, *P*< 0.001).

### Relationship between plasma HMGB1 level and parameters of metabolism and inflammation was stated in Table 2

**Table 2:** Partial correlations analysis of variables associated with circulating HMGB1 concentration in study population.

	Plasma HMGB1		Plasma HMGB1 (age- and sex-adjusted)		Plasma HMGB1 (age-, sex- and BMI adjusted)	
variables	r	*p*	r	*p*	r	*p*
Age(year)	0.335	<0.001	-	-	-	-
BMI	0.777	<0.001	0.419	<0.001	-	-
WHR	0.450	<0.001	0.317	<0.001	0.310	<0.001
SBP	0.278	<0.001	0.289	<0.001	0.166	0.041
DBP	0.263	0.001	0.207	0.010	0.214	0.008
FPG	0.280	<0.001	0.212	0.008	0.104	0.037
2hPG	0.229	0.004	0.159	0.050	0.035	0.067
HbA_1C_	0.244	0.002	0.379	<0.001	0.132	0.104
FINS	0.360	<0.001	0.417	<0.001	0.127	0.018
HOMA-IR	0.447	<0.001	-0.393	<0.001	0.264	0.024
HOMA-β	-0.399	<0.001	0.184	0.023	-0.132	0.039
Cre	0.156	0.053	-0.244	0.002	0.055	0.504
HDL-c	-0.232	0.004	0.039	0.631	-0.160	0.049
LDL-c	0.052	0.519	0.028	0.734	0.111	0.175
TC	0.013	0.875	0.257	0.001	0.104	0.204
TG	0.170	0.030	0.443	<0.001	0.220	0.006
IL-6	0.494	<0.001	0.792	<0.001	0.193	0.017

BMI, body mass index; Wc, waist circumference; WHR, waist hip ratio; SBP, systolic blood pressure; DBP, diastolic blood pressure; FPG, fasting plasma glucose; 2hPG, 2h postchallenge plasma glucose; HbA_1C_, glycated hemoglobin; FINS, fasting serum insulin; HOMA-IR, Homeostasis Model Assessment for insulin resistance; HOMA-β, Homeostasis Model Assessment for beta-cell function; TC, total cholesterol; TG, triglyceride; HDL-c, high-density lipoprotein-cholesterol; LDL-c, low-density lipoprotein-cholesterol; IL-6, interleukin- 6.

The levels of plasma HMGB1 were positively correlated with WHR, SBP, DBP, FPG, FINS, HOMA-IR, TG, IL-6, and negatively correlated with HOMA-β and HDL-c. All the correlations remained statistically significant after adjustment for age, gender and BMI.

### Associations of circulating HMGB1 levels with diabetes in fully adjusted models were stated in Table 3

**Table 3:** Associations of circulating HMGB1 levels with diabetes in full adjusted models.

Model adjustment	Diabetes		
	OR	95% CI	*P*
Non-adjusted	2.256	1.748–2.553	0.001
Age	2.224	1.706–2.497	<0.001
Age,Sex	2.217	1.695–2.428	0.001
Age,Sex,SBP	2.179	1.670–2.314	<0.001
Age,Sex,SBP,DBP	2.125	1.674–2.278	<0.001
Age,Sex,SBP,DBP,TG, TC, HDL, LDL	1.875	1.732–2.146	<0.001
Age,Sex,SBP,DBP,TG, TC, HDL, LDL,Cre	1.862	1.730–2.019	<0.001
Age,Sex,SBP,DBP,TG, TC, HDL, LDL,Cre,BMI	1.764	1.533–1.878	<0.001

OR, odds ratio; CI, confidence interval; BMI, body mass index;SBP, systolic blood pressure; DBP, diastolic blood pressure;TC, total cholesterol; TG, triglyceride; HDL-c, high-density lipoprotein-cholesterol; LDL-c, low-density lipoprotein-cholesterol.

Multivariate logistic regression analyze revealed that plasma HMGB1 levels were significantly correlated with diabetes even after controlling for age, sex, blood pressure, lipid profile, Cre and BMI. (All *P*<0.01).

## Discussion

HMGB1 was initially classified asa nuclear protein that regulates transcription,and itis a dominant member ofthe High-Mobility Group (HMG) family, which are identified as low molecular weight, non-histone chromosomal proteins[7,8]. Conventionally, the main functions of nuclear HMGB1 are transcriptional regulation and modulation of chromosomal architecture. It can stabilize chromosomesbybending DNA and facilitating the binding of several regulatory proteins such as the nuclear hormone-receptor family, V(D)J recombinases and p53–p73 transcriptional complexes to DNA[9–13]. In 1999, cytosolic HMGB1 was discovered to be a proinflammatory mediator that is secreted by activated macrophages in response to injury, infection or other inflammatory stimuli [14]. As was mentioned, chronic low-grade inflammation (LGI)is a central link between T2DM and obesity, and previous studies indicated that HMGB1 was strongly related with inflammation[2–4]. Therefore, the clinical significance of HMGB1 deserves to be explored in obesity and T2DM patients. In the current study, we detected plasma HMGB1 concentrations in those patients and investigated its relationship with relevant factors.

Previous studies had explored several possible mechanisms forthe induction of plasma inflammation by HMGB1. There are currently three definite signaling pathways that regulate the proinflammatory effects of plasma HMGB1. The first pathway isHMGB1 binding tothe advanced glycation endproduct (RAGE) receptor [15]. The combination of HMGB1 and RAGE can regulate cell motility and neurite outgrowth[16]. However, in metabolic disease, it mainly activatesNuclear factor κB (NFκB), a classical inflammatory mediator[17]. Interestingly, HMGB1 binding to RAGE can upregulate RAGE signaling, thus creates a positive feedback loop to amplify inflammation[18]. The second pathway is binding to Toll-like receptor 2 (TLR2). The third pathway is binding to TLR4. The primary roles of these two pathways are triggering NFκB to modulate systemic inflammatory responses[19]. As a well-known inflammatory mediator, activated NFκB stimulates the biosynthesis of various proinflammatory mediators in cells such as tumor necrosis factor-α (TNF-α), interleukin-6 (IL-6) and IL-1β, which participate the pathological inflammatory response [20]. Meanwhile, Brice et al. observed that plasma HMGB1 could bind to RAGE and boost IL-6 expression in preadipocytes and contribute to LGI implementation and maintenance[21]. It is now clearly established that IL-6 can regulate inflammation to modulate glucose metabolism. Elevated hepatic IL-6 levelscan induce chronic inflammation to cause IR and promote diabetes. Conversely, IL-6 promotes LGI to impair insulin signaling and weaken insulin sensitivity in visceral fat [22]. Thus, we chose IL-6 to reflect the inflammation status of obese and T2DM patients. Simultaneously, increasing basic and clinical researchverifiedthe proinflammatory function of HMGB1. Devaraj et al. observed that ligands of TLR2 and TLR4(including HMGB1) were highly expressed either in monocytes of patients with type 1 diabetes (T1DM) or in serum from newly diagnosed T2DM subjects[23–24]. Manoj et al. demonstrated that HMGB1 is more highly expressed in adipose tissue from obese patients than control patients, which is attributed to inflammation[25].

In the current study, we observed that HMGB1 levels in the OB group were significantly higher than those in the NW group (Fig 1). Additionally, correlation analysis also demonstrated that plasma HMGB1 levels were positively correlated with body fat parameters such as BMI and WHR even after adjustment for age and gender (Table 2). These results indicate that plasma HMGB1 was strongly correlated with obesity independent of age and gender, which is consistent with the conclusions of previous studies[4]. Moreover, plasma HMGB1 levels of the T2DM-NW group were increased compared with the NGT-NW group. This finding might demonstrate that dysglycemia could also increase HMGB1 in addition to obesity.

BecauseLGI is importantin the progression of both obesity and T2DM, we surmisedthat the underlying mechanism ofincreasing of hyperglycemia-induced HMGB1 expression was mainly related toinsulin resistance (IR). IR, a main cause for hyperinsulinemia, is a common state in diabetes. In this study, we observed that FINS levels and HOMA-IR in the T2DM group were increased more than the NGT group (Table 1),and plasma HMGB1 concentrations were positively correlated with HOMA-IR (Table 2), which was consistent with the opinion above. It was unequivocally demonstratedby several studies that hyperinsulinemia could regulate the synthesis of various cytokines[26–29]. Therefore, it is reasonable to speculate that the IR-induced hyperinsulinemia could contribute to modulate HMGB1 production. The research of Guzmánfurther confirmed the relationship between IR and HMGB1 by demonstrating that circulating HMGB1 levels were increased in adipocytes from IR subjects. Meanwhile, they observed that serum HMGB1 levels could accelerate insulin release and intracellular Ca(2+) concentration in INS-1 cells[30]. Ni et al. demonstrated that plasma HMGB1 was increased in women with polycystic ovary syndrome (PCOS) who had intense IR and hyperinsulinemia[31]. Several studies also indicated that HMGB1 could influence β-pancreatic cell function. Lee et al. observed that INS-1 cell apoptosis was increased by after1 μg/ml HMGB1 treatment, which might have been mediated by oxidative stress[32]. Our current data that plasma HMGB1 levels wereassociated with diabetes independent of BMI further confirmed this phenomenon (Table 3).

Moreover,in our study, plasma HMGB1 levels were positively correlated with triglyceride (TG) levels (Table 2), which suggested that HMGB1 might influence lipid metabolism. Dao et al. determined that atorvastatin could reduce the total cholesterol (TC) and low-density lipoprotein-cholesterol (LDL-c) levels by decreasing plasma HMGB1 concentrations[33]. In addition to atorvastatin, several compounds such as glucocorticoids and chloroquine also attenuated serum HMGB1secretion[34,35]. It is worth mentioning that glucocorticoid regulation of HMGB1-mediated inflammation is mainly driven by the TLR4 pathway[36].

However, our study has some limitations that should be noted. First, the sample size of our study wasquite small, thus,research including a large number of subjects needs to be implemented. Second, we didnot consider the influence of race, so it is necessary to measure the plasma HMGB1 levels in persons of other ethnicities. Last but not least, our study is cross-sectional, so we cannot explain the causality between plasma HMGB1 levels and T2DM or obesity.

In summary, we detected HMGB1 serum levels in patients with T2DM and obesity or both. We found circulating HMGB1 concentrations were significantly increased not only in obeseindividualsbut also inpureT2DM patients. Extracellular HMGB1 as a cytokine is highly correlated with parameters of obesity, IR and inflammation. Therefore, it may indicate and be involved in the pathology of obesity and T2DM.

## Supporting Information

S1 Table Correlations analysis of variables associated with circulating HMGB1 concentration in subgroups. (DOCX)
