# Supplementary material for: Plasma HMGB-1 Levels in Subjects with Obesity and Type 2 Diabetes: A Cross-Sectional Study in China
Source: PLoS One. 2015 Aug 28;10(8):e0136564. doi: 10.1371/journal.pone.0136564 (PMC4552731; doi:10.1371/journal.pone.0136564)
Supplement: S1 Table — (DOCX) [file pone.0136564.s001.docx]

Supplement Table 1. Correlations analysis of variables associated with circulating HMGB1 concentration in subgroups.

|  | Plasma HMGB1 | | Plasma HMGB1 | | Plasma HMGB1 | | Plasma HMGB1 | |
| --- | --- | --- | --- | --- | --- | --- | --- | --- |
| variables | (NGT-NW) | | (NGT-OB) | | (T2DM-NW) | | (T2DM-OB) | |
|  | r | *P* | r | *P* | r | *P* | r | *P* |
| Age(year) | 0.030 | 0.865 | 0.276 | 0.085 | 0.111 | 0.519 | 0.355 | 0.25 |
| BMI | 0.401 | 0.026^*^ | 0.248 | 0.021^*^ | 0.376 | 0.016^*^ | 0.240 | 0.040^*^ |
| WHR | 0.362 | 0.017^*^ | 0.387 | 0.003^**^ | 0.219 | 0.059 | 0.331 | 0.048^*^ |
| SBP | 0.103 | 0.533 | 0.047 | 0.774 | 0.119 | 0.490 | 0.085 | 0.603 |
| DBP | 0.023 | 0.889 | 0.076 | 0.639 | 0.075 | 0.663 | 0.046 | 0.780 |
| FPG | 0.333 | 0.038^*^ | 0.311 | 0.050^*^ | 0.272 | 0.008^**^ | 0.285 | 0.024^*^ |
| 2hPG | 0.114 | 0.488 | 0.192 | 0.236 | 0.060 | 0.728 | 0.341 | 0.031^*^ |
| HbA_1C_ | 0.016 | 0.924 | 0.326 | 0.040^*^ | 0.065 | 0.760 | 0.210 | 0.192 |
| FINS | 0.206 | 0.208 | 0.396 | 0.011^*^ | 0.327 | 0.006^**^ | 0.043 | 0.001^*^ |
| HOMA-IR | 0.263 | 0.106 | 0.211 | 0.019^*^ | 0.260 | 0.029^*^ | 0.238 | 0.013^*^ |
| HOMA-β | -0.299 | 0.064 | -0.037 | 0.052 | -0.189 | 0.027^*^ | -0.273 | 0.018^*^ |
| Cre | 0.301 | 0.063 | 0.022 | 0.892 | 0.193 | 0.259 | 0.108 | 0.509 |
| HDL-c | -0.219 | 0.181 | -0.061 | 0.709 | -0.006 | 0.972 | -0.021 | 0.898 |
| LDL-c | 0.086 | 0.604 | 0.053 | 0.748 | 0.025 | 0.885 | 0.114 | 0.485 |
| TC | 0.051 | 0.756 | 0.104 | 0.524 | 0.026 | 0.880 | 0.151 | 0.351 |
| TG | 0.332 | 0.039^*^ | 0.413 | 0.008^**^ | 0.191 | 0.263 | 0.111 | 0.495 |
| IL-6 | 0.211 | 0.042^*^ | 0.307 | 0.027^*^ | 0.604 | 0.018^*^ | 0.564 | 0.009^**^ |

NGT, normal glucose tolerance; T2DM, type 2 diabetes mellitus; NW, normal weight; OB, obesity; BMI, body mass index; Wc, waist circumference; WHR, waist hip ratio; SBP, systolic blood pressure; DBP, diastolic blood pressure; FPG, fasting plasma glucose; 2hPG, 2 hours postchallenge plasma glucose; HbA_1C_, glycated hemoglobin ; FINS, fasting serum insulin; HOMA-IR, Homeostasis Model Assessment for insulin resistance; HOMA-β, Homeostasis Model Assessment for beta-cell function; TC, total cholesterol; TG, triglyceride; HDL-c, high-density lipoprotein-cholesterol; LDL-c, low-density lipoprotein-cholesterol; IL-6, interleukin- 6. ^*^*P* < 0.05, ^**^*P* < 0.01.
